# Dépression et niveau de fardeau chez les aidants familiaux des sujets déments en Tunisie

**Published:** 2011-11-23

**Authors:** Jihène Ben Thabet, Feriel Jaoua, Nada Charfi, Lobna Zouari, Nasreddine Zouari, Mohamed Maalej

**Affiliations:** 1 Service de Psychiatrie C, CHU Hédi Chaker, 3029 Sfax, Faculté de médecine de Sfax, Université de Sfax, Tunisie

**Keywords:** Démence, aidants familiaux, fardeau, dépression

## Abstract

**Introduction:**

La démence peut retentir lourdement sur les aidants familiaux du patient. Les objectifs de notre étude étaient de déterminer le niveau de fardeau et de la dépression chez les aidants familiaux de sujets déments, et d'identifier les facteurs associés à un niveau de fardeau élevé.

**Méthodes:**

Il s'agissait d'une enquête auprès de 65 aidants tunisiens. Les niveaux de fardeau et de la dépression ont été évalués par, respectivement, l'inventaire de Zarit et l’échelle de Beck.

**Résultats:**

Le taux des aidants qui avaient un niveau de fardeau élevé était de 52,3 %. Une dépression modérée ou sévère a été relevée chez 46,2 %. Un niveau de fardeau élevé était corrélé, du côté de l'aidant, avec le niveau socio-économique moyen à élevé, la cohabitation avec le patient, le fait d’être son conjoint, la réduction des activités quotidiennes et la sévérité de la dépression, et, du côté du dément, avec l'agressivité.

**Conclusion:**

Les facteurs corrélés à un niveau de fardeau élevé orientent vers les cibles d'intervention et sont susceptibles d’être améliorés par la prise en charge, ce qui contribuerait à alléger la détresse des aidants.

## Introduction

La démence altère non seulement la qualité de vie des patients, mais aussi retentit lourdement sur les aidants. De nombreux travaux soulignent les répercussions négatives de cette aide sur la santé des aidants, en particulier leur santé mentale [1]. L'effet des différents facteurs de stress sur ces aidants représente le fardeau ou la charge [2]. La fonction d'aide peut être influencée par des facteurs culturels et religieux.

La Tunisie est en situation interculturelle. Pays arabo-musulman, certes, mais aussi au voisinage de l′Europe et aux portes de l′Afrique. Aussi, la société tunisienne est-elle en pleine mouvance. Elle vit de nombreux contrastes du fait des deux influences: arabo-musulmane, plutôt conservatrice, accordant un statut privilégié au sujet âgé, et occidentale, beaucoup plus libérale. Cette atmosphère, contraignante et contradictoire, pourrait avoir des répercussions sur la fonction d'aide et la charge ressentie.

Nos objectifs étaient de dépister la dépression chez les aidants familiaux de personnes démentes et d’évaluer leur niveau de fardeau et l′impact du facteur culturel sur le niveau de fardeau perçu.

## Méthodes

Il s'agissait d'une étude transversale, qui a consisté en une enquête menée auprès des aidants familiaux tunisiens de sujets déments. Pour optimiser la représentativité de notre échantillon, les sujets déments ont été recrutés à la fois dans le secteur public (à l'unité des consultations externes du service de psychiatrie au CHU Hédi Chaker), à Sfax en Tunisie, et dans les cabinets de cinq psychiatres exerçant dans le secteur privé dans la même région. Tous les aidants qui ont accompagné leur parent dément à des consultations psychiatriques entre août 2009 et février 2010 ont été sollicités. Deux avaient refusé de participer à l'enquête. Les aidants qui ont accepté de participer à l'enquête ont répondu à :

Un questionnaire anonyme concernant des donnés sociodémographiques, des caractéristiques de la situation d'aide (lien de parenté, cohabitation, recours aux aides professionnelles et à domicile, réduction des activités de l'aidant) ainsi que des données médicales concernant le malade (durée d’évolution de la maladie, notion de prise de traitement et sa durée, sévérité de l'atteinte cognitive évaluée à l'aide du Mini Mental State Examination (MMSE), alimentation, temps des repas, hygiène, présence de troubles du comportement, agressivité envers l'aidant).

L'inventaire de fardeau de Zarit (pour évaluer le fardeau): Pour chacun des 22 items de cette échelle, l′aidant a déterminé à quelle fréquence il ressentait telle ou telle émotion dans sa relation d′aide. Cette fréquence pouvant varier de 0 « jamais » à 4 « presque toujours ». Un score global, obtenu en faisant la somme des scores de chacun des items, peut varier théoriquement de 0 à 88. Plus le score est é1evé, plus la charge ressentie est importante: le fardeau est léger entre 21 et 40, modéré entre 41 et 60 et sévère au-delà de 61 [3,4]. Conformément à la littérature [3], nous avons considéré la médiane du score du fardeau, obtenu dans notre étude, comme valeur approximative du seuil d'un niveau de fardeau élevé (lourdement ressenti).

L’échelle d'auto-évaluation de Beck (pour dépister la dépression), comprenant 13 items coté chacun de 0 à 3. Un score global, obtenu en faisant la somme des scores de chacun des items, peut varier théoriquement de 0 à 39: la dépression est légère entre 4 et 7, modérée entre 8 et 15 et sévère au-delà de16.

L’étude a comporté deux parties: une descriptive et une analytique. L'analyse statistique des données a été réalisée par le logiciel SPSS dans sa quinzième version. L’étude comparative s'est basée sur le Test Chi-deux. Le seuil de significativité retenu a été fixé à 5%.

## Résultats

Le nombre d′aidants colligés était de 65.

### Etude descriptive

Caractéristiques médicales des déments

L′âge moyen des sujets déments de notre série était de 75 ± 6,86 ans, avec des extrêmes de 60 et 97 ans. Le sexe ratio (H/F) était de 0,75. La durée d’évolution de la maladie de nos patients était en moyenne de 3 ± 2,83 ans avec une durée moyenne de prise en charge des sujets déments de 3 ± 2,25 ans (Tableau 1).

**Tableau 1:** Caractéristiques médicales des déments

Variables	Nombre	Pourcentage (%)
**Score à la MMSE**		
Modéré	25	38,5
Sévère	40	61,5
**Alimentation**		
Seul	29	44,6
Avec aide	36	55,4
**Temps des repas**		
Long	41	63,1
Normal	24	36,9
**Hygiène**		
Assurée seul	6	9,2
Assurée par l'aidant	59	90,8
**Troubles du comportement**		
Oui	50	76,9
Non	15	23,1
**Agressivité envers l'aidant**		
Oui	42	64,6
Non	23	35,4

Caractéristiques sociodémographiques des aidants et celles relatives à la situation d'aide

L′âge moyen des aidants de notre série était de 49 ± 11,13 ans, avec des extrêmes de 20 et 73 ans. Le sexe ratio (H/F) était de 0,18 (Tableau 2).

**Tableau 2:** Caractéristiques sociodémographiques des aidants et celles relatives à la situation d'aide

Variables	Nombre (N)	Pourcentage (%)
**Niveau socio-économique**		
Bas	21	32,3
Moyen	19	44,6
Elevé	15	23,1
**Résidence**		
Rurale	21	32,3
Urbaine	44	67,7
**Lien de parenté**		
Conjoint	14	21,5
Descendant ou fratrie du dément	51	78,5
**Lieu de vie**		
Avec le dément	38	58,5
Différent du dément	27	41,5
**Aide à domicile**		
Oui	22	33,8
Non	43	66,2
**Aide médicale**		
Oui	22	33,8
Non	43	66,2
**Réduction des activités de l'aidant**		
Oui	58	89,2
Non	7	10,8
**Sensation de fardeau**		
Oui	46	70,8
Non	19	29,2

Description du fardeau

Les résultats concernant le sentiment de fardeau sont résumés dans le Tableau 3. La moyenne du fardeau dans notre échantillon était de 42 ±18,37 avec des extrêmes de 11 et 88. La médiane du score du fardeau étant 42, 34 aidants (52,3 %) avaient un fardeau élevé et 31 (47,7 %) avaient un fardeau léger.

**Tableau 3:** Répartition des aidants selon le score de l'inventaire de fardeau de Zarit

	Nombre (N)	Pourcentage (%)
Fardeau nul	8	12,3
Fardeau léger	21	32,3
Fardeau modéré	22	33,8
Fardeau sévère	14	21,6

La médiane du score du fardeau, considérée comme valeur approximative du seuil d'un fardeau élevé, était égale à 42. Ainsi, 34 aidants (52,3 %) avaient un fardeau élevé et 31 (47,7 %) avaient un fardeau léger.

Dépistage de la dépression

La moyenne du score de Beck était de 4,87 ± 4,2 avec des extrêmes de 0 et 15 (Tableau 4).

**Tableau 4:** Répartition des aidants selon le score de l’échelle de Beck

	Nombre (N)	Pourcentage (%)
Pas de dépression	24	36,9
Dépression légère	11	16,9
Dépression modérée	18	27,7
Dépression sévère	12	18,5

### Etude analytique

Un niveau de fardeau élevé était corrélé, dans notre étude, de façon significative aux variables suivantes (Tableau 5): le niveau socio-économique de l'aidant, le lien de parenté (conjoint du dément), le lieu de vie partagé avec le dément, l'alimentation avec aide du dément, la réduction des activités quotidiennes, l'existence d'agressivité envers l'aidant, la sensation de fardeau exprimée par l'aidant et un score de Beck d'au moins 10,54.

**Tableau 5:** Corrélations des variables sociodémographiques et cliniques avec le niveau du fardeau

	Fardeau bas		Fardeau élevé		p

	N	%	N	%	
**Sexe de l'aidant**					
Masculin	3	30	7	70	0,2
Féminin	28	50,9	27	49,1	
**Niveau socio-économique de l'aidant**					
Bas	14	66,7	7	33,3	0,034
Moyen à Elevé	17	38,7	27	61,3	
**Parenté avec le dément**					
Conjoint	3	21,4	11	78,6	0,026
Enfant	28	54,9	23	45,1	
**Lieu de vie**					
Avec dément	12	31,5	26	68,5	0,002
Distinct de celui du dément	19	70 ,4	8	29,6	
**Aide à domicile**					
Oui	12	54,5	10	45,5	0,42
Non	19	44,1	24	55,9	
**Aides médicales**					
Oui	10	45,5	12	54,5	0,79
Non	21	48,8	22	51,2	
**Alimentation**					
Seul	18	62,1	11	37,9	0,037
Aidé	13	36,1	23	63,9	
**Temps du repas**					
Long	19	46,3	22	53,7	0,77
Normal	12	50	12	50	
**Hygiène**					
Seul	4	66,7	2	33,3	0,32
Aidé	27	45,8	32	54,2	
**Agressivité du dément**					
Oui	15	35,7	27	64,3	0,009
Non	16	69,6	7	30,4	
**Sensation de fardeau**					
Oui	16	34,8	30	65,2	0,001
Non	15	78,9	4	21,1	
**Réduction des activités**					
Oui	24	41,4	34	58,6	0,003
Non	7	100	0	0	
**Sévérité de la dépression**					
Pas de dépression ou dépression légère	22	62,9	13	37,1	0,001
Dépression modérée à sévère	7	23,3	23	67,7	

Il n'y avait pas de corrélation statistique avec les variables suivantes: le sexe de l'aidant, la sévérité de l'atteinte cognitive du patient (estimée par le score à la MMSE), le degré d'autonomie du dément (hygiène), et le fait de bénéficier d′une aide médicale ou à domicile.

## Discussion

### Description du fardeau

Dans notre étude, le niveau de fardeau moyen était de 42. Ce score correspond à la limite supérieure de ceux rapportés dans la littérature (entre 22,4 et 42) [3–7]. Cette variabilité serait sous tendue par deux facteurs :

Plus la fonction d′aide assumée par l′aidant se prolonge dans le temps, plus la sensation de fardeau est lourde. L'intégration, dans les échantillons étudiés, des aidants accompagnant un proche depuis plusieurs années (3±2,25 ans dans notre étude, 4,4±2,5 ans dans la série de Kerhervé [4]) contribue incontestablement à élever le niveau de fardeau ressenti avec des moyennes respectives de 42 et 38,7. D'ailleurs, dans notre étude, le niveau de fardeau augmentait de façon significative avec la durée de prise en charge du dément (p= 0,02).

Des méthodologies différentes: le niveau de fardeau est plus bas pour les enquêtes réalisées en population générale que celles sélectionnées par l'intermédiaire de services de soins (consultation externe de psychiatrie et cabinets de psychiatres de libre pratique). En effet, en population générale, très peu de déments sont traités pour leur pathologie démentielle et dans un nombre non négligeable de cas, on peut supposer que la maladie n′est connue ni du patient ni de son entourage [8].

### Fardeau et dépression

Le score moyen de l’échelle de Beck (8,33±7,05), chez les aidants de notre étude, était supérieur à celui de la population générale (7±7,05) [2]. Le taux des aidants de notre étude qui présentaient une dépression modérée à sévère (46,2 %) était supérieur à celui observé dans l’étude de Kerhervé (22,8 %) [4]. Pour ces aidants, une prise en charge en consultation externe de psychiatrie a été suggérée.

Les scores à l'inventaire du fardeau de Zarit et à l’échelle de dépression de Beck étaient fortement corrélés dans notre étude (p = 0,001); ce qui rejoint les résultats de l’étude de Kerhervé [4]. Toutefois, il est difficile de faire la part entre l′effet d'une dépression préexistante, ou d'une récidive chez des sujets ayant des antécédents dépressifs (non pris en compte dans notre recueil des données) et la dépression d′épuisement en rapport avec une charge plutôt lourde.

### Facteurs associés à un niveau de fardeau élevé

Parmi les facteurs corrélés à un niveau de fardeau élevé, chez les aidants de notre étude, figuraient avec le niveau socio-économique de l'aidant (p=0,034), le lien de parenté avec le dément (p=0,026), le lieu de vie partagé avec le dément (p=0,002), l'alimentation avec aide du dément (p=0,037), la réduction des activités quotidiennes (p=0,003), l'existence d'agressivité (physique et verbale) envers l'aidant (p=0,009) et la sensation d'un niveau de fardeau élevé chez l'aidant (p=0,001).

Sexe de l′aidant

De nombreuses études montrent que les femmes rapportent un niveau de fardeau ou un niveau de tension plus important que celui des hommes. Cette prédominance féminine aurait de nombreuses explications. Les femmes auraient une implication affective plus importante et une moindre inhibition à exprimer une plainte ou un symptôme. Elles sont non seulement plus fréquemment sollicitées pour effectuer des tâches d′aide, mais aussi elles bénéficient moins de support social dans cette fonction [9]; elles auraient tendance à être moins souvent secondées dans leur activité d′aide par leur entourage que les aidants de sexe masculin [10]. Alors qu'en fait, prendre en charge une personne adulte n′est pas naturel pour la femme; cette tâche serait plus stressante pour elle que pour l'homme, lequel peut prendre des décisions plus facilement. Enfin, cette activité d′aide survient à un âge où la femme a fini d′élever ses enfants et pourrait avoir du temps disponible pour ses loisirs [11].

Dans notre échantillon qui comportait majoritairement des femmes (84,6%), le sexe n'intervenait pas dans le degré du fardeau, contrairement aux données de la littérature [3,4]. En effet, dans notre contexte socioculturel et religieux, le rôle essentiel de la femme est de subvenir aux besoins de son foyer et sa famille, y compris ses parents et/ou beaux-parents à un âge plus avancé. Cette tâche fait donc partie du rôle social de la femme qu'elle assume sans s'en plaindre.

Niveau socio-économique de l'aidant

Dans notre étude, il y avait une corrélation entre un niveau de fardeau élevé et un niveau socio-économique moyen à élevé des aidants. Ce résultat était vraisemblablement biaisé, compte tenu de la composition de notre population d’étude qui comportait majoritairement des femmes d'origine urbaine et d'un niveau socioéconomique moyen à élevé (63,6% des femmes), profil très différent de celui classique de la femme tunisienne sus-décrite. Celles-là sont dans une situation d′entre-deux culturel, tiraillées entre des racines arabo-musulmanes conservatrices et des influences modernes et libérales. Ce contexte socio-économique va de pair avec un cumul de charges professionnelles et familiales, entre autres celle de la tâche d'aide. Le stress lié à un tel contexte familial et socioprofessionnel constitue un facteur de vulnérabilité à l’épuisement (burn-out), à un sentiment de niveau de fardeau élevé et à la dépression.

L'agressivité

Dans la plupart des travaux, la charge ressentie par l′aidant est plus liée aux troubles du comportement qu′aux incapacités pour effectuer les actes élémentaires de la vie quotidienne [12]. Les troubles du comportement de l′aidé sont mal vécus par l′aidant, de façon générale, et, selon l’étude Rabins [3], la violence physique est le trouble du comportement le plus difficile à gérer.

Dans notre étude, il y avait une corrélation entre un niveau de fardeau élevé et l'agressivité (physique ou verbale) de l'aidé envers l'aidant; ce qui rejoint les données de la littérature [3].

Le lien de parenté

Pour les aidants de notre étude, une charge élevée a été plus souvent rapportée par les conjoints que par les enfants des sujets déments (78,6% versus 45,1%).

Dans notre contexte arabo-musulman, l'enfant est tenu d'avoir du respect vis-à-vis de ses parents, d'accepter leurs décisions et, si besoin, de les prendre en charge sur les plans matériel et physique quel que soit le contexte. Ces valeurs, fondamentales dans notre culture, sont inculquées aux enfants dès leur jeune âge; ce qui rendrait la fonction d'aide plus facile à gérer pour ces derniers que pour les conjoints qui sont parfois des épouses ayant enduré durant leur vie conjugale l'autoritarisme, voire la maltraitance de leur mari.

### Le recours aux aides médicales et à domicile

**Le recours aux aides médicales** est associé à un fardeau é1evé dans de nombreuses études [3] et est certainement le témoin de problèmes de santé. En effet, il semble que la sévérité de l'atteinte cognitive, les troubles du comportement, l'altération du statut nutritionnel et, par conséquent, le recours aux aides médicales soient intriqués. Pour les aidants de notre étude, bien qu'ils fussent majoritairement d'un niveau socio-économique moyen ou élevé, le recours à ce type d'aide pouvait être perçu comme un rejet condamnable par la société (notamment le recours au placement dans des institutions spécialisées). Les aidants préfèreraient alors gérer seuls les problèmes de prise en charge de leurs proches déments.

Quant au **recours aux aides à domicile**, les études, y compris la nôtre, ne montrent pas d'association avec un niveau de fardeau plus bas [3,4]. Si les services d′aides à domicile soutiennent l′aidant dans la gestion des déficiences instrumentales de la personne aidée, ils restent peut-être insuffisants pour la prise en charge d′une personne démente. En effet, la prise en charge d′une personne présentant une altération des fonctions supérieures nécessite une surveillance continue, peu compatible avec l′activité des services d′aides à domicile [3].

### Limites de notre travail

Les principales limites de notre étude étaient: le nombre peu élevé d'aidants enquêtés; l'inventaire de fardeau de Zarit utilisé dans notre travail mesure uniquement la dimension négative du fardeau et ne prend pas en considération les aspects positifs de l'aide, comme la valorisation de la personne aidante et le renforcement des liens entre l'aidant et l'aidé, qui ont une valeur adaptative [3,4]. En outre, il ne permet pas d'identifier avec précision les besoins des aidants en souffrance [4].

## Conclusion

Nos résultats reflètent la détresse des aidants familiaux des sujets déments. Les facteurs corrélés à un niveau de fardeau élevé sont, en partie, des facteurs non modifiables qui peuvent cependant nous permettre de cibler une population à risque susceptible de bénéficier d'une attention particulière. Certains facteurs modifiables pourraient faire l'objet d'une prise en charge adaptée, et ceci dès l'apparition des premiers symptômes, afin de soulager les aidants. De tels travaux devraient être complétés par des études longitudinales du fardeau, afin de repérer les facteurs prédictifs d'un fardeau é1evé. Ainsi, il sera possible d'identifier des aidants vulnérables devant bénéficier de mesures d'aides adaptées.
